# Digital patient modeling identifies predictive biomarkers of regorafenib response in elderly metastatic colorectal cancer

**DOI:** 10.3389/fsysb.2025.1648559

**Published:** 2025-09-15

**Authors:** Juan Manuel García-Illarramendi, Pedro Matos-Filipe, Jose Manuel Mas, Judith Farrés, Xavier Daura

**Affiliations:** ^1^ Anaxomics Biotech S.L., Barcelona, Spain; ^2^ Institute of Biotechnology and Biomedicine, Universitat Autònoma de Barcelona, Barcelona, Spain; ^3^ Research Programme on Biomedical Informatics (GRIB), Department of Experimental and Health Science, Universitat Pompeu Fabra, Barcelona, Spain; ^4^ Catalan Institution for Research and Advanced Studies (ICREA), Barcelona, Spain; ^5^ Centro de Investigación Biomédica en Red de Bioingeniería, Biomateriales y Nanomedicina, Instituto de Salud Carlos III, Cerdanyola del Vallès, Spain

**Keywords:** *In silico* clinical trial, metastatic colorectal cancer, machine learning, regorafenib, transcriptomics data, predictive biomarkers

## Abstract

*In* *silico* clinical trials that simulate individualized mechanisms of action offer a powerful approach to assess drug efficacy across large and diverse patient populations, while also enabling the identification of predictive biomarkers. In this study, we conducted an *in silico* clinical trial of first-line, single-agent regorafenib in 399 elderly patients with metastatic colorectal cancer (mCRC). Individualized network-based models were constructed using patient-specific differential transcriptomic profiles and employed to simulate the target-specific effects of regorafenib. From this analysis, we identified both predictive and mechanistic biomarkers of treatment response. Notably, four proteins—MARK3, RBCK1, LHCGR, and HSF1—emerged as dual biomarkers, showing associations with both response mechanisms and predictive potential. Three of these (MARK3, RBCK1, and HSF1) were validated in an independent cohort of mCRC patients and were also found to be targets of previously reported regorafenib-predictive miRNAs. This study demonstrates a novel systems biology strategy for evaluating drug response *in silico*, leveraging transcriptomic data to simulate individual treatment outcomes and uncover clinically relevant biomarkers. Our findings suggest that such approaches may serve as valuable complements to traditional clinical trials for assessing drug efficacy and guiding precision oncology.

## 1 Introduction

Computational modeling and simulation have long played a key role across various stages of drug development, from target discovery to clinical evaluation (Lesage et al., 2023; Sadybekov and Katritch, 2023). In the context of clinical studies, early efforts primarily focused on pharmacokinetic and pharmacodynamic models. However, as our understanding of the molecular basis of disease has advanced, mechanism-based approaches—also known as systems biology models—have gained prominence. These models integrate biological pathways and processes to more accurately simulate disease progression and therapeutic effects (Ayyar and Jusko, 2020).

These advances have laid the foundation for the development of *in silico* clinical trials (ISCTs) (Pappalardo et al., 2019), which use virtual patient populations and biological simulations to emulate real-world clinical trials. ISCTs offer a rational, hypothesis-driven framework for exploring treatment efficacy (Arulraj et al., 2024) identifying biomarkers (Subudhi et al., 2022) and informing clinical trial design (Creemers et al., 2023; Voutouri et al., 2024). Importantly, they also allow researchers to assess risks and biases in trial protocols prior to their implementation, ultimately supporting the development of safer and more effective therapies.

A variety of modeling strategies and virtual patient generation methods have been developed, each with unique strengths. Some approaches generate “virtual patients” using population-level data (Coto-Segura et al., 2023; Creemers et al., 2023), while others generate “digital twins or digital patients” based on individual-level omics (Kalari et al., 2018) and clinical data (Wang et al., 2024). Patient-specific models constructed from transcriptomic or proteomic profiles can capture inter-individual variability in disease mechanisms and treatment responses and provide a more accurate representation of real-world data.

Oncology is among the therapeutic areas where *in silico* methodologies have seen the most widespread application. Within this field, metastatic colorectal cancer (mCRC) remains a significant clinical challenge due to its high incidence, aggressive course, and poor prognosis—particularly among elderly patients (Bray et al., 2018). While early-stage colorectal cancer has a favorable 5-year survival rate exceeding 90%, median survival drops to approximately 2 years in the metastatic setting (Bradley et al., 2011; Franchi et al., 2019). Notably, individuals aged 65 years and older make up more than two-thirds of the mCRC population and account for the majority of disease-related mortality (Ferlay et al., 2015). Despite numerous approved treatment options, only two molecular biomarkers—RAS mutation status (predictive for anti-EGFR therapies) and microsatellite instability (MSI) status (predictive for immunotherapy)—are currently implemented in clinical practice. However, MSI-high (MSI-H) tumors represent only a small fraction of CRC cases, and even within this subgroup, response to immunotherapy is not guaranteed (Hou et al., 2022). Regorafenib, a multikinase inhibitor, has demonstrated encouraging disease control and overall survival in elderly mCRC patients, supporting its potential utility as a first-line treatment in this subgroup. However, no validated biomarkers are currently available to predict which patients will benefit most from this therapy (Carrato et al., 2019). This underlines an urgent and unmet need for novel predictive biomarkers that can guide treatment selection and enable more effective patient stratification and personalized care in metastatic colorectal cancer.

In this study, we present an *in silico* clinical trial evaluating first-line single-agent regorafenib in elderly mCRC patients. We employ a systems biology modeling platform (Jorba et al., 2020; Segu-Verges et al., 2021), which uses a neural network-like algorithm to propagate biological signals through the human protein network. Patient-specific models are constructed from transcriptomic profiles, enabling the simulation of individualized mechanisms of action (MoA) and drug responses. Our aim is to identify both predictive and mechanistic biomarkers of regorafenib efficacy. The findings are further validated in an independent patient cohort and linked to a previously established panel of regorafenib-associated predictive miRNAs.

## 2 Materials and methods

### 2.1 *In silico* clinical trial study

Our analysis emulates a clinical trial with individual mCRC patient models assessing the action of regorafenib. The simulation incorporates multiple interconnected components defined below.

#### 2.1.1 Model stimulus and mCRC molecular definition

The model is stimulated by inhibiting 18 known protein targets of regorafenib with pharmacological activity, as reported in DrugBank version 5.1.19 (Wishart et al., 2018) (see Supplementary Table S1).

A molecular definition of mCRC was obtained from the Biological Effector Database (BED) (Jorba et al., 2020), which is based on manual curation of scientific literature. In BED, proteins are annotated according to their contribution to disease: activation-associated proteins receive a score of +1, and inhibition-associated proteins receive a score of −1. The mCRC protein knowledge set used in this study as outset for the models (see Supplementary Table S2) includes 236 proteins.

#### 2.1.2 IDE generation

Gene expression data for mCRC were obtained from the GEO database (Edgar et al., 2002; Barrett et al., 2013). We identified 485 mCRC samples from colorectal biopsies of untreated patients aged 70–88 years, forming the discovery population. Of these, 77 samples were excluded due to missing raw microarray data or prior normalization adjustments (e.g., quantile normalization, surrogate variable adjustment), leaving 408 mCRC patient samples for analysis (see Supplementary Table S3). In parallel, we retrieved 49 healthy control samples from the same GEO experiments (see Supplementary Table S4). CuBlock cross-platform normalization (Junet et al., 2021) was applied to all samples at the probe level. Probe expression values were then converted to protein expression by averaging the expression of all probes mapping to each protein.

RNA-seq gene expression data was obtained from The Cancer Genome Atlas (TCGA) for a validation cohort of 67 mCRC patients, selected to match the age range of the discovery cohort. Additionally, data from 23 healthy colon or rectum tissue samples were included for comparison. Prior to conversion to protein expression levels, expression data from both mCRC and healthy samples were normalized using the Trimmed Mean of M-values method.

Normal protein expression ranges were established from the expression distribution of healthy samples. Following the approach of Kalari et al. (2018), proteins in mCRC samples with expression values above the 95th percentile or below the 5th percentile of the expression distribution observed in healthy samples were considered upregulated (+1) or downregulated (−1), respectively. Proteins with expression values within this range were considered normally expressed and excluded from further analysis. This process generated individual differential expression (IDE) signatures for each of the 408 mCRC samples.

To refine the IDEs, we identified differentially expressed genes (DEGs) through population-level analysis. DEGs between pooled mCRC and healthy samples were identified with Welch’s t-test and *DESeq2* (Love et al., 2014) with a false discovery rate (FDR) from two-sided p values of less than 0.05 for the discovery and validation mCRC populations, respectively. We then selected proteins that were within three interaction links of the mCRC protein knowledge set in our protein interaction network. Proteins in the IDE signatures that did not appear in both the DEG list and the network proximity list were excluded from the final IDEs.

#### 2.1.3 TPMS modelling

The methodology applied in this study has been previously described (Jorba et al., 2020; Gutierrez-Casares et al., 2021). In brief, the Therapeutic Performance Mapping System (TPMS) is a systems biology-based approach that constructs mathematical models of mechanisms of action (MoAs) to explain the relationship between a biological stimulus and a clinical response.

TPMS models are built upon the Human Protein Network (HPN), a comprehensive map of human proteins and their known interactions. These include physical interactions, signaling and metabolic pathways, and gene regulation mechanisms, integrated from curated databases such as KEGG (Kanehisa et al., 2025), REACTOME (Milacic et al., 2024), intACT (Del Toro et al., 2022), BIOGRID (Oughtred et al., 2021), HPRD (Peri et al., 2004), and TRRUST (Han et al., 2015). TPMS uses sampling-based methods to generate models analogous to multilayer perceptrons, where proteins represent nodes and known interactions define edges. Upon perturbation of the HPN with a stimulus, the signal propagates through connected nodes based on the activation or inhibition state of each protein and the directionality and weight of their interactions. This propagation occurs over three iterative steps. In the first step, the input signal is transmitted from the drug target nodes to their directly connected neighbors. Each receiving node integrates the input signals from its upstream nodes, with each contribution weighted according to the corresponding edge weight (representing interaction strength and direction). The sum of these inputs is then transformed using a hyperbolic tangent function, which normalizes the signal and limits its magnitude. In the second step, the newly activated nodes pass their output signals to their own downstream neighbors, again weighted by edge strength and passed through the same transformation. In the third and final step, this propagation continues once more to the next layer of directly connected nodes. The cumulative effect of these three iterations allows the signal to reach biologically relevant downstream response proteins while preventing over-diffusion of the perturbation signal across the network thereby generating plausible MoAs. Model training incorporates general constraints (e.g., known drug–indication pairs) and user-defined specific conditions. The accuracy of each model is evaluated by the percentage of constraint fulfillment. Network parameters (edge weights) are optimized via a stochastic optimization method based on simulated annealing, which adjusts interaction strength and direction based on probabilistic measures derived from biological evidence. Due to the underdetermined nature of the system—where the number of training constraints is typically smaller than the number of model parameters—TPMS yields a population of feasible solutions.

In this study, the stimulus is defined as the inhibition of regorafenib drug targets, while the response set comprises proteins from mCRC knowledge set. The objective is to propagate the signal from the inhibited targets to the response proteins in a manner that reverses their pathological activity, as previously defined. Each TPMS solution is characterized by a distinct pattern of protein activity across the HPN. The final model is derived by averaging across all valid solutions with most solutions clustering around a dominant mechanistic pattern. This observation is supported by the low uncertainty scores computed for our TPMS models. Specifically, the uncertainty score quantifies solution variability as the ratio between the observed Shannon entropy of the ensemble and the maximum possible entropy for the model. In the case of the regorafenib simulation, the median uncertainty score across models was 20.5%, indicating relatively high convergence among solutions. Given this low variability, averaging the signal across all valid solutions was considered appropriate and biologically meaningful, as it captures the most robust and recurrent mechanistic patterns while minimizing the influence of outliers. Proteins with a final non-zero signal value between −1 and +1 are considered active downstream proteins. The *in silico* response is quantified using a score called TSignal, calculated as an average of the activity values of response proteins (mCRC molecular definition set). Activities in the corresponding sum will be positive when the protein shows its predefined expected modulation (e.g., if we simulate a drug treatment, we expect a reversal of the pathological signal) and negative if it deviates from its expected direction. The summatory is then divided by the total number of proteins reached. Correctly modulated proteins contribute positively to TSignal, while incorrectly affected proteins reduce the score. The TSignal of a TPMS model was calculated as the mean TSignal of the solutions.

#### 2.1.4 Calibration of *in silico* signal to clinical outcomes

To improve the clinical relevance of our *in silico* response metric, TSignal, we implemented a calibration step to align TPMS-predicted responses with real-world clinical outcomes. We selected five drugs approved for first-line treatment of mCRC, for which overall survival (OS) data were available from published clinical trials: cetuximab (Sastre et al., 2011), regorafenib (Carrato et al., 2019), bevacizumab (Cunningham et al., 2013), capecitabine (Cunningham et al., 2013) and irinotecan (Sanli et al., 2006). To estimate the survival benefit attributable to each treatment, we subtracted OS data—obtained from placebo-treated populations in previously published studies (Scheithauer et al., 1993; Ho et al., 2005) — from the reported OS for each drug. Where possible, we matched placebo OS data to the trial population’s characteristics (e.g., age, treatment setting) to account for inter-study variability (see Supplementary Table S5).

TPMS models were generated for each drug by setting the drug’s known targets as the stimulus and the full mCRC protein knowledge set as the response. For each model, we computed the corresponding TSignal value and assessed its association with clinical benefit by calculating the Pearson correlation between TSignal and the reported OS for each drug. To optimize this relationship, we applied an iterative feature selection process, whereby proteins were progressively removed from the response list if their exclusion improved the correlation between TSignal and OS. This stepwise refinement was repeated until the maximum Pearson correlation was achieved. The final calibrated subset comprised 233 proteins (see Supplementary Table S2), resulting in a Pearson correlation coefficient of 0.87 between TSignal and OS across the five drug models (see Supplementary Figure S1). This optimized protein set was used for all subsequent TPMS analyses.

#### 2.1.5 Individual model construction using TPMS

The construction of individual patient models using the Therapeutic Performance Mapping System (TPMS) follows a two-step simulation process, designed to capture both the molecular characteristics of the disease state and the patient-specific response to treatment. Both steps along with the calibration of the *in silico* signal are run in MATLAB (MathWorks, 2024).

In the first step, TPMS simulates the disease state of each patient based on their IDE profile, which represents the molecular condition prior to treatment. To initiate the simulation, a set of 20 proteins located in proximity to mCRC knowledge set was selected as the stimulus. The signal originating from these proteins is propagated through the network with the aim of maintaining the disease-representative signal across the mCRC knowledge set, which acts as the response. Patient-specific IDEs were incorporated as restrictions during model training. These restrictions served two purposes: they promoted the inclusion of IDE proteins in the network by retrieving connections between them and the defined stimulus and response nodes, and they increased the model’s accuracy when IDE proteins were present in the final solution with the correct activation or inhibition sign. A solution was considered valid if at least 50% of the IDE proteins present had the correct sign and at least 60% of the proteins in the mCRC knowledge set were reached by the propagated signal. For each patient, 50 valid MoA solutions were retained, representing the initial disease state.

In the second step, the effect of regorafenib treatment was simulated by applying the drug’s inhibitory effect to the MoA solutions generated in the disease state modeling. The goal of this simulation was to revert the pathological activity of the proteins in the mCRC knowledge set. As in the first step, a solution was considered valid if at least 60% of the mCRC knowledge set proteins were reached. However, in this phase, the activity signs of IDE proteins were allowed to change, and there was no constraint on the number of IDE proteins included in the final solution. For each patient, 50 valid treatment-state MoA solutions were collected, which represent the individualized response model to regorafenib. The thresholds used in the modeling steps were empirically determined based on prior experience with the TPMS framework, representing a practical compromise that preserves biological interpretability while ensuring feasibility across the dataset.

### 2.2 Identification of mechanistic and predictive biomarkers

Mechanistic biomarkers of regorafenib response were identified by comparing MoA proteins of good and poor *in silico* responders within the discovery mCRC population. Proteins with an absolute mean activation difference greater than 0.1 and an FDR <0.05, based on Welch’s t-test, were considered significant. In the validation cohort, due to its smaller sample size, mechanistic biomarkers were identified using the Mann–Whitney *U* test. Proteins with a median absolute activation difference >0.1 and a *p*-value <0.05 were deemed significant.

Similarly, predictive biomarkers were identified by comparing the frequency and sign of inclusion of IDE proteins between good and poor *in silico* responders using Fisher’s exact test. A *p*-value <0.05 was used as the threshold for significance.

Enrichment analyses of the mechanistic biomarkers identified in the discovery cohort were done using the KEGG (Kanehisa et al., 2025) and REACTOME (Milacic et al., 2024) pathway annotations. Statistical significance was assessed using the hypergeometric test, with the background limited to all proteins with predicted activity across the full set of MoAs. Pathways were considered significantly enriched if they had a false discovery rate (FDR) <0.05 and included at least 10% of the input protein set.

All statistical and enrichment analyses were conducted using R statistical software (v4.2.2) (R Core Team, 2025), *stats* base R package and the *clusterProfiler* R package (Xu et al., 2024).

### 2.3 Association between miRNAs and proteins

A panel of 12 microRNAs (miRNAs)—hsa-miR-126-3p, hsa-miR-126-5p, hsa-miR-139-5p, hsa-miR-140-3p, hsa-miR-143-5p, hsa-miR-152-3p, hsa-miR-185-5p, hsa-miR-28-3p, hsa-miR-338-3p, hsa-miR-362-3p, hsa-miR-551, and hsa-miR-582-5p—was previously identified as predictive of response to first-line single-agent regorafenib treatment (Conde et al., 2021). Protein targets for these miRNAs were retrieved from miRGate (Andrés-León et al., 2015), which integrates both experimentally validated and computationally predicted interactions. Validated targets were obtained from MiRTarBase (Huang et al., 2022), miRecords (Xiao et al., 2009) and OncomirDB (Wang et al., 2014). Predicted targets were sourced from five different databases: miRanda (Enright et al., 2003), Pita (Kertesz et al., 2007), RNAHybrid (Rehmsmeier et al., 2004), Microtar (Thadani and Tammi, 2006) and TargetScan (McGeary et al., 2019). miRNA–protein interactions were considered more reliable if they were experimentally validated or, in the case of predicted targets, if they were supported by at least three independent databases.

### 2.4 Assessing the predictive value of mechanistic and predictive biomarkers

The predictive ability of 3 proteins—HSF1, MARK3 and RBCK1—to *in silico* regorafenib response was assessed with the CuBlock normalized expression of the identified good and poor responders in univariate logistic regression models. The area under the receiver operator curve (AUC) after 10-fold cross-validation (CV) with 100 repetitions was computed for each protein. A CV-AUC >0.7 was considered as significant to determine their predictive ability (Hosmer et al., 2013).

## 3 Results

**FIGURE 1:**
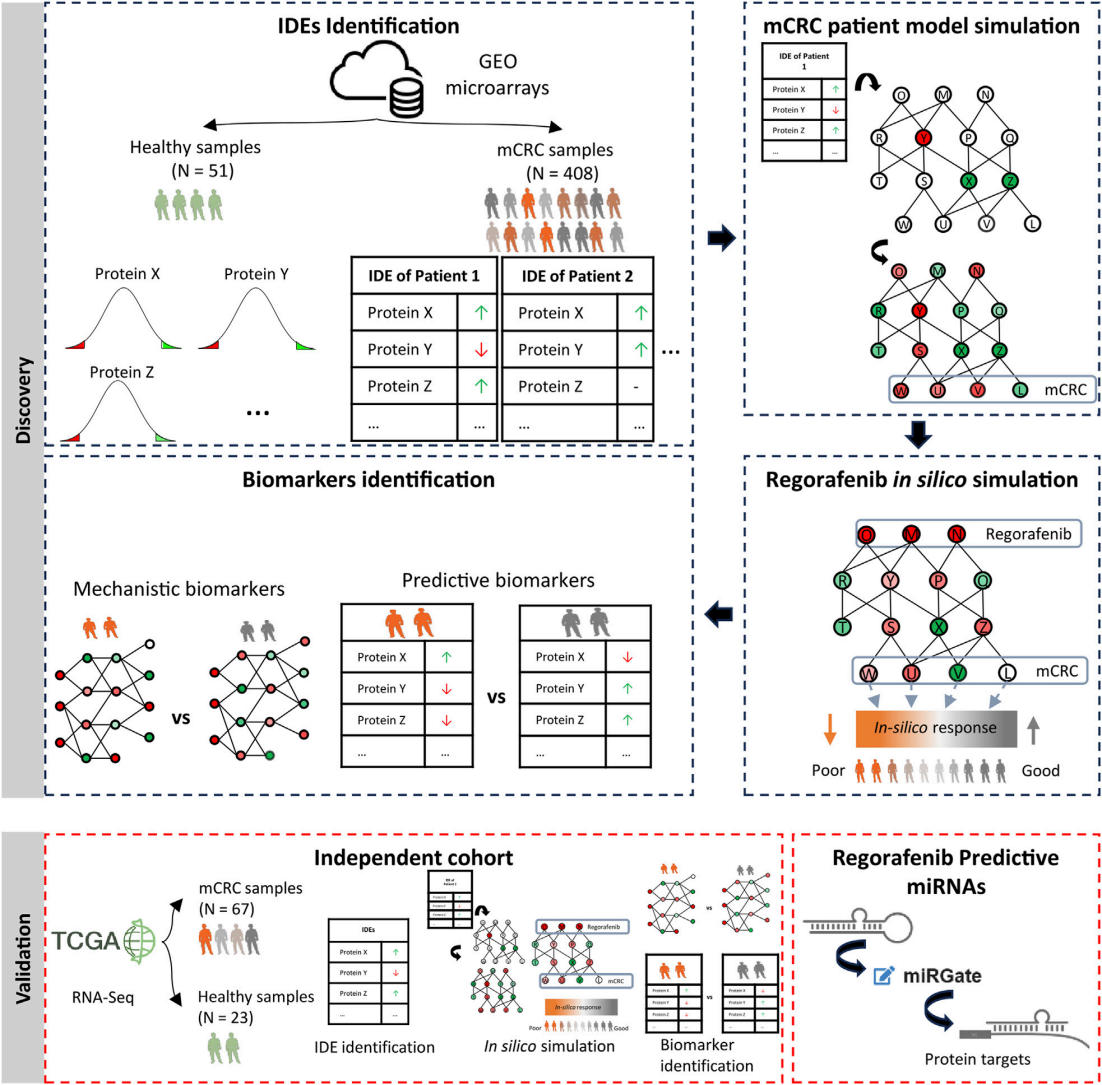
Schematic overview of the study workflow and key steps. Discovery phase with individual differential proteins identification (IDEs), individual model building and simulation, and biomarkers identification. Validation phase with study replication in an independent cohort and linking to a previously established panel of regorafenib-associated predictive miRNAs.

This study was conducted in two main phases (see Figure 1). In the discovery phase, a large cohort of digital patient models was generated using individual transcriptomic profiles, followed by *in silico* simulation of regorafenib treatment to identify mechanistic and predictive biomarkers of response. In the validation phase, the same modeling and simulation procedures were applied to an independent patient cohort to confirm the robustness of the findings. Additionally, the identified predictive biomarkers were evaluated for their association with a previously established panel of regorafenib-related predictive miRNAs.

### 3.1 Patient-specific differential expression profiles in elderly mCRC

The target population for our study was elderly patients with untreated metastatic colorectal cancer (mCRC) receiving first-line treatment with single-agent regorafenib. To build individual *in silico* models, we derived individual differentially expressed genes (IDEs) for 408 mCRC patients using valid microarray data obtained from the GEO database.

IDEs were defined as proteins with expression values above the 95th percentile or below the 5th percentile of the distribution observed in healthy control samples. This method typically results in a large number of differentially expressed proteins per patient (mean IDE proteins per patient: 626). To enhance the specificity and biological relevance of these profiles, we applied a two-step refinement process. First, we identified 3,342 proteins (see Supplementary Table S6) that were significantly differentially expressed between the pooled mCRC and healthy samples, population DEGs. All of these genes appeared in at least one of the individual IDE profiles, confirming that population mCRC dysregulation is preserved within the individual-level models. Second, we restricted the IDEs to include only proteins located within three interaction links of the mCRC protein knowledge set; 10,176 proteins, in our network model. This step ensured that each IDE consisted of proteins not only differentially expressed but also mechanistically relevant to mCRC pathogenesis. Six out of the 408 patients were excluded from the analysis due to having a low number of IDE proteins. The resulting IDE profiles are thus patient-specific, yet rooted in population-level disease features and constrained by network-based proximity to mCRC knowledge set. Each patient’s profile reflects a distinct combination of disease-associated protein alterations (see Supplementary Table S7).

### 3.2 Simulation of first-line single-agent regorafenib and definition of good and poor responders

In a simulated clinical trial framework, individualized response models to regorafenib were constructed using a two-step simulation process. In the first step, each patient’s identified IDEs were used to construct an initial disease-state MoA model. These models then served as the basis for simulating the effect of regorafenib treatment in the second step.

**FIGURE 2:**
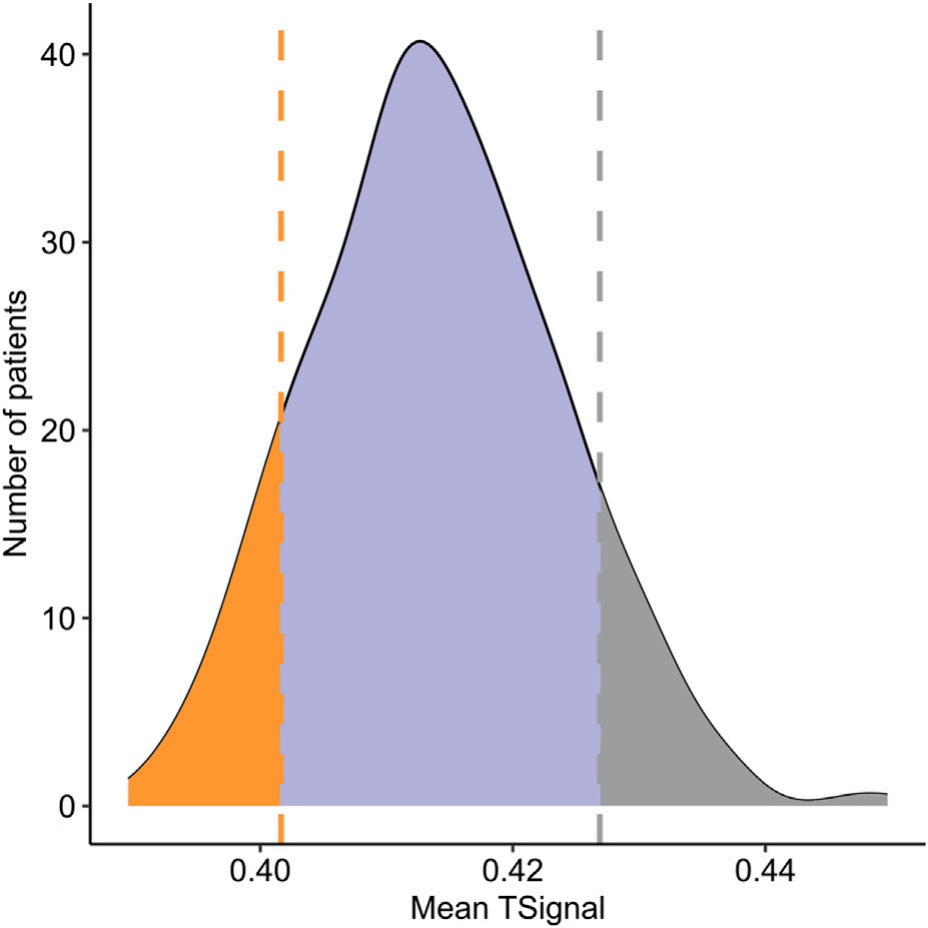
Distribution of *in silico* response scores (TSignal) across 399 metastatic colorectal cancer (mCRC) digital patients in the discovery cohort. The TSignal represents the predicted response to regorafenib. Horizontal lines mark the 10th and 90th percentiles, used as thresholds to define *in silico* poor (orange) and good (grey) responders, respectively.

For three patients, no valid solutions were found during the initial disease-state modeling, and they were therefore excluded from further analysis. In total, 399 individualized regorafenib treatment models were successfully generated. The extent of drug impact on the 233-proteins mCRC knowledge set—used to represent the functional landscape of disease biology—was quantified using the *in silico* signal metric (TSignal) that has been adjusted to better align with actual clinical outcomes (see Supplementary Figure S1). This value reflects the predicted ability of regorafenib to reverse pathological protein activity. The distribution of the TSignal across the 399 mCRC models followed a normal distribution (see Figure 2).

To explore potential mechanistic and predictive differences between good and poor *in silico* responders, we stratified patients into two groups: good responders, defined as those with mean TSignal values above the 90th percentile (n = 40), and poor responders, defined as those below the 10th percentile (n = 40). These thresholds correspond to estimated OS values of 6.95 and 7.79 months, respectively.

No statistically significant differences were observed in age, sex and cancer stage between the population of good and poor *in silico* regorafenib responders (see Table 1).

**TABLE 1:** Demographic and clinical characteristics of the discovery mCRC population, including a comparison between the subgroups of good and poor *in silico* responders identified through treatment simulation.

Discovery mCRC population	All	In silico response subsets		
Characteristic	N = 399^a^	Poor N = 40^a^	Good N = 40^a^	p-value^b^
Age	77.0 (73.0–81.0)	75.5 (72.0–78.5)	77.0 (73.5–80.5)	0.4
Sex				>0.9
Female	182 (46%)	22 (55%)	22 (55%)	
Male	217 (54%)	18 (45%)	18 (45%)	
Cancer stage (AJCC)				0.8
III	253 (72%)	27 (75%)	26 (70%)	
IV	99 (28%)	9 (25%)	11 (30%)	
Unknown	47	4	3	

^a^ Median (Q1 - Q3); n (%).

^b^ Welch’s Two Sample t-test; Fisher’s exact test.

### 3.3 Mechanistic and predictive biomarkers identification

The stratification of patients into mechanistic good and poor responders to regorafenib enabled the identification of two types of potential biomarkers: mechanistic biomarkers, derived from the comparison of MoAs, and predictive biomarkers, derived from the comparison of IDEs.

**FIGURE 3:**
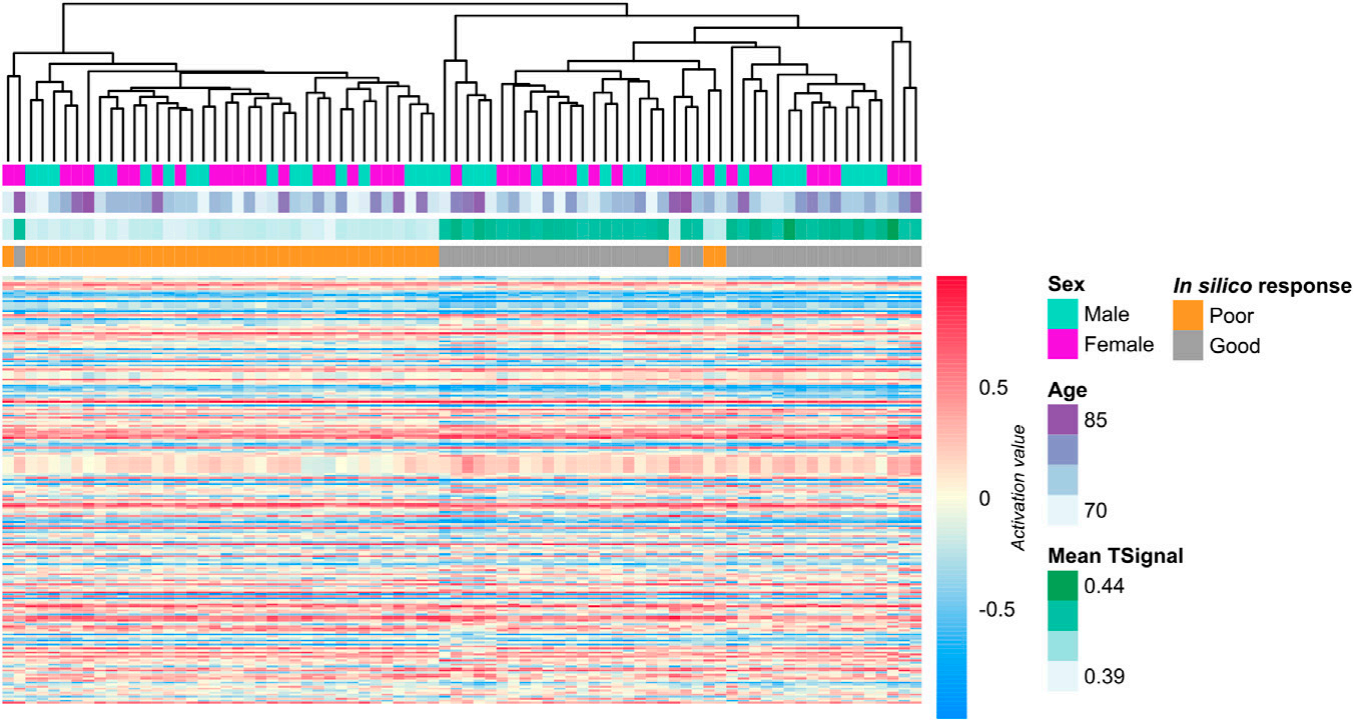
Heatmap of mechanistic biomarkers associated with regorafenib response in the discovery mCRC cohort. Rows represent the 213 mechanistic biomarkers identified, and columns represent individual digital patients, clustered based on predicted protein activity profiles. Annotations indicate each patient’s predicted *in silico* response category, biological sex, age, and model-derived TSignal value.

Mechanistic biomarkers were identified by comparing protein activation levels in the MoAs between good and poor responders. A total of 213 proteins showed statistically significant differential activity (Welch’s t-test, adjusted *p*-value <0.05; mean absolute difference >0.1; see Supplementary Table S8). Unsupervised clustering based on the activity of these mechanistic proteins clearly distinguished good from poor responders, with only one good responder clustering with poor responders and 3 poor responders clustering with the good responders (see Figure 3). Enrichment analysis of these proteins showed a significant presence of proteins more active in good responders involved in “[R-HSA-73894] DNA Repair”. While proteins with higher activity in poor responders showed enrichments in “04151_PI3K-Akt signaling pathway”, “04621_NOD-like receptor signaling pathway”, “04620_Toll-like receptor signaling pathway”, “05131_Shigellosis”, “05162_Measles” and “[R-HSA-1643685] Disease” (see Supplementary Table S9).

In parallel, a comparison of IDE profiles between good and poor responders yielded 173 proteins with significantly different frequencies, representing potential predictive biomarkers (Fisher’s exact test, *p*-value <0.05; see Supplementary Table S10). The enrichment analysis of this predictive protein did not reveal any significantly enriched KEGG and REACTOME pathways.

The two sets of biomarkers—mechanistic and predictive—were found to be highly interconnected in the HPN used by TPMS, with many proteins linked through direct interactions (see Supplementary Figure S2). Notably, four proteins—LHCGR, MARK3, HSF1, and RBCK1—were common to both sets, highlighting their potential biological relevance.

**FIGURE 4:**
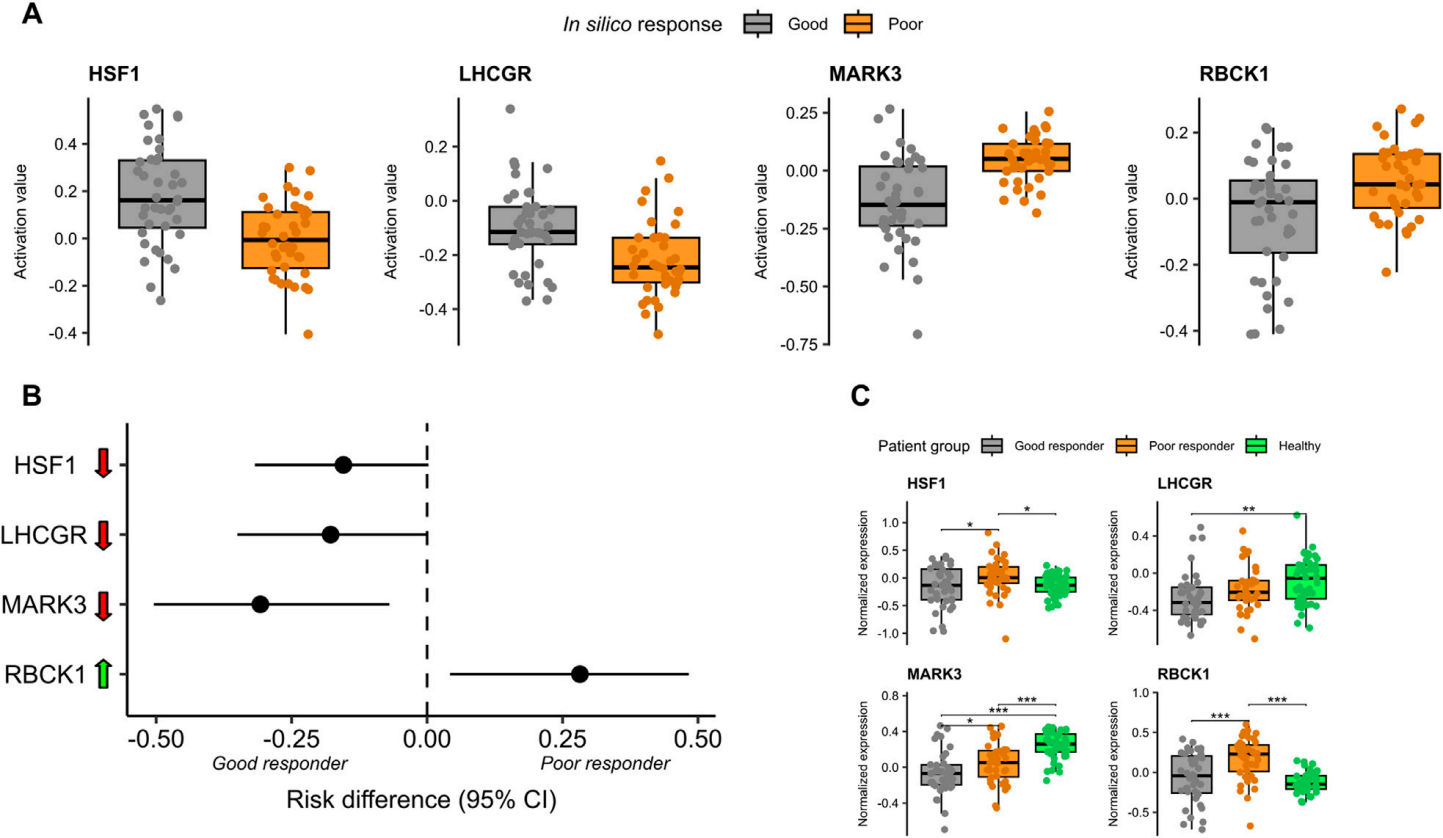
Mechanistic and predictive biomarkers associated with regorafenib response in the discovery mCRC cohort. **(A)** Boxplots showing predicted protein activity levels of HSF1, LHCGR, MARK3, and RBCK1 in *in silico* good and poor responders. **(B)** Risk differences calculated using Newcombe’s method, representing the difference in the proportion of patients with ↓ under- or ↑ overexpression of each biomarker in the individual differential expression (IDE) profiles. **(C)** Boxplots of normalized protein expression levels for the four biomarkers across good responders, poor responders, and healthy control samples.

HSF1, LHCGR, and MARK3 exhibited reduced expression in the IDEs of good responders, meaning their expression levels were lower than those observed in healthy controls. In contrast, RBCK1 was overexpressed in the IDEs of poor responders compared to control levels (see Figure 4B). This expression pattern was also reflected in the normalized transcriptomic data; however, statistically significant differences relative to healthy controls were observed only for LHCGR, MARK3, and RBCK1 (see Figure 4C).

Simulation of regorafenib action revealed that in good responder models, HSF1 and LHCGR showed increased activity post-treatment, suggesting a reversal of their initially low activation states. However, MARK3 and RBCK1 maintained their baseline activation patterns following treatment: MARK3 remained less active in good responders, and RBCK1 more active in poor responders (see Figure 4A).

### 3.4 Independent cohort validation

To validate our findings, we replicated the entire analysis in an independent cohort of 67 mCRC patients with similar demographic characteristics to the discovery population (see Table 2). Gene expression data in form of RNA-Seq were obtained from TCGA, along with 23 healthy colon or rectal tissue samples from CRC patients (see Supplementary Table S11).

**TABLE 2:** Demographic and clinical characteristics of the validation mCRC population, including a comparison between the subgroups of good and poor *in silico* responders identified through treatment simulation.

Validation mCRC population	All	In silico response subsets		
Characteristic	N = 67^a^	Poor N = 5^a^	Good N = 17^a^	p-value^b^
Age	77.0 (73.0–81.0)	75.0 (73.0–82.0)	75.0 (71.0–80.0)	0.3
Sex				>0.9
Female	32 (48%)	2 (40%)	9 (53%)	
Male	35 (52%)	3 (60%)	8 (47%)	
Cancer stage (AJCC)				>0.9
III	41 (61%)	3 (60%)	9 (53%)	
IV	26 (39%)	2 (40%)	8 (47%)	

^a^ Median (Q1 - Q3); n (%).

^b^ Mann-Whitney U test; Fisher’s exact test.

Following the same TPMS simulation procedure used in the discovery phase, we identified IDEs for each of the 67 mCRC patients and generated individualized regorafenib treatment models. Patients were then classified as good responders (17 patients) or poor responders (5 patients) using the same TSignal thresholds that defined good and poor responders in the discovery cohort. No significant differences in the demographic and clinical variables were observed between the good and poor *in silico* responders in this validation cohort (see Table 2).

Comparison of MoAs between good and poor responders in this validation cohort identified 211 proteins (see Supplementary Table S12) with significantly different activity, 64 of which overlapped with the mechanistic biomarkers from the discovery cohort (see Supplementary Figure S3), Similarly, comparing IDE profiles between good and poor responders yielded 79 proteins (see Supplementary Table S13) with significantly different frequencies. Five of these proteins were also identified as predictive biomarkers in the discovery cohort analysis (see Supplementary Figure S3).

**FIGURE 5:**
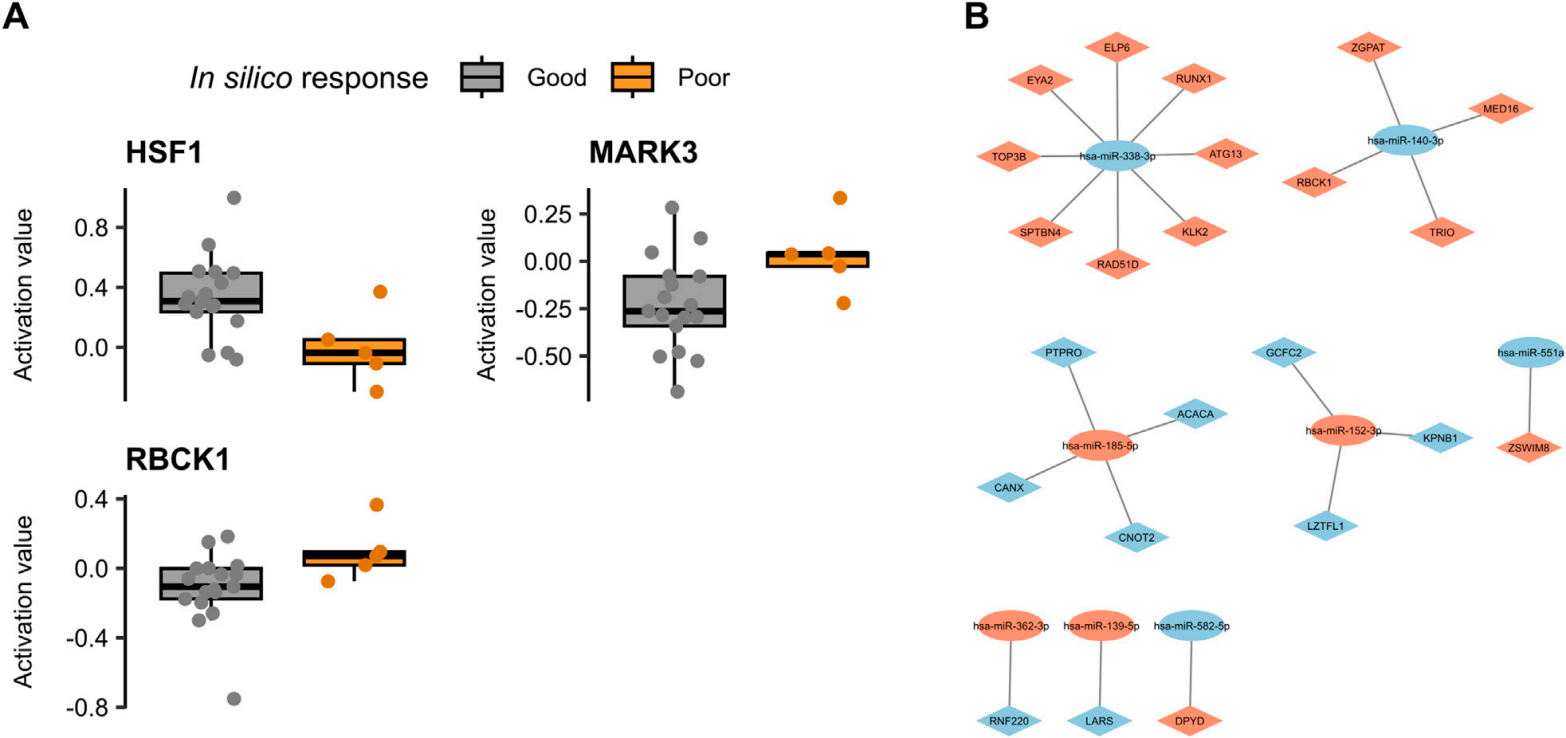
Validation of the mechanistic and predictive biomarker identified in the discovery mCRC population. **(A)** Boxplots of predicted protein activity levels for HSF1, MARK3, and RBCK1 in *in silico* good and poor responders within the validation mCRC cohort. **(B)** Network diagram showing associations between previously identified dysregulated predictive miRNAs (oval nodes) and predictive biomarkers from the discovery cohort (diamond-shaped nodes). Node color indicates expression direction in poor responders: orange for upregulated, blue for downregulated.

Of the four top candidate biomarkers identified in the discovery phase (MARK3, RBCK1, LHCGR, and HSF1), three—MARK3, RBCK1, and HSF1—were successfully validated as mechanistic biomarkers in the independent validation cohort. These proteins showed consistent activation profiles in the regorafenib simulations: HSF1 exhibited higher activity in good responder models compared to poor responders, while MARK3 and RBCK1 remained less active in good responders compared to poor responders (Figure 5A).

### 3.5 Validation of predictive biomarkers through previously identified regorafenib-associated miRNAs

A panel of 12 miRNAs has previously been identified as predictive of response to first-line single-agent regorafenib treatment (Conde et al., 2021). To explore potential links between these miRNAs and the predictive biomarkers identified in our study, we assessed whether any of the 178 predictive biomarkers were known targets of the 12 miRNAs, based on established miRNA–protein interactions.

Across all interaction types (including both predicted and experimentally validated), we found that 167 of the 173 predictive biomarkers of the discovery cohort had at least one known association with the miRNA panel (see Supplementary Table S14). When restricting the analysis to higher-confidence interactions—i.e., those that were experimentally validated or predicted by at least three independent algorithms—we identified 46 predictive proteins as reliable targets of the predictive miRNAs. Of these 46 proteins, 23 exhibited expression patterns consistent with the regulatory direction of the associated 9 miRNAs. Specifically, proteins linked to miRNAs that were overexpressed in good responders showed lower protein expression in those patients, and *vice versa* (Figure 5B).

Notably, three of our most promising predictive biomarkers—MARK3, HSF1, and RBCK1—were among the proteins targeted by these miRNAs. However, only RBCK1 had a high-confidence interaction with one of the predictive miRNAs (hsa-miR-140-3p) and consistent regulatory direction, strengthening its potential role as a clinically relevant biomarker.

### 3.6 Assessing the predictive value of mechanistic and predictive biomarkers

We evaluated the predictive potential of the three biomarkers—HSF1, MARK3, and RBCK1—which were identified as both predictive and mechanistic in the discovery cohort, and validated in the independent validation cohort. All three proteins showed significant differences in normalized expression between the 40 good and 40 poor responders in the discovery cohort (Welch’s t-test, FDR <0.05), with lower expression levels associated with good responders (Figure 4C).

**FIGURE 6:**
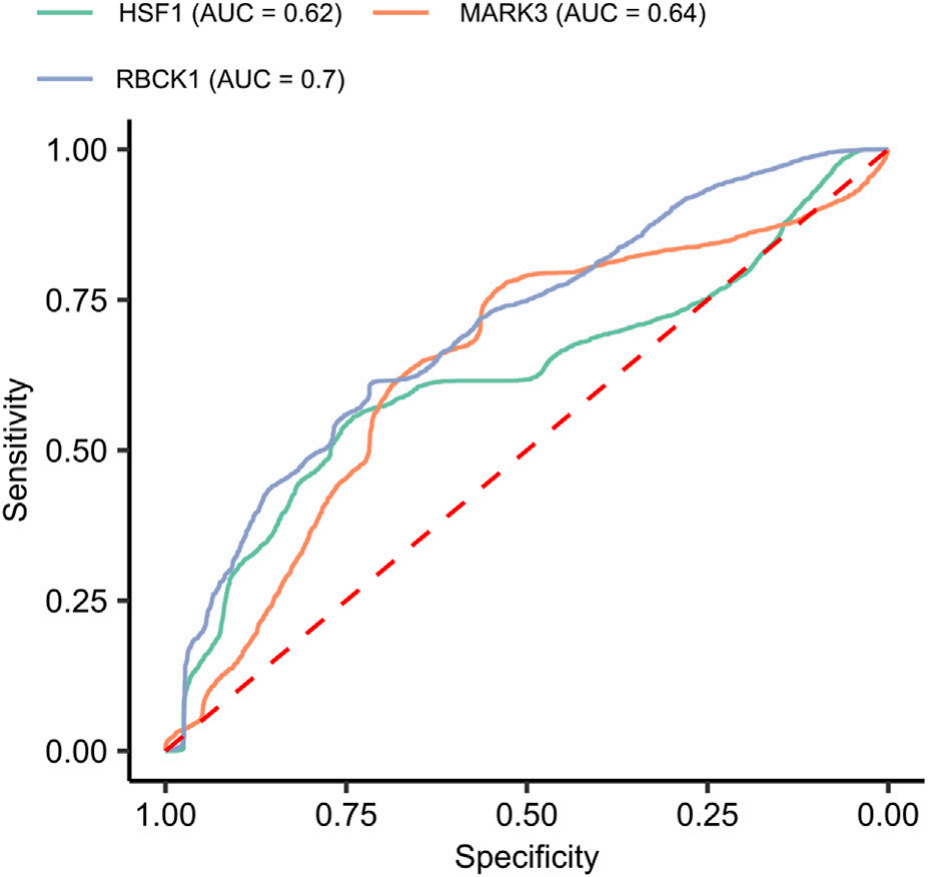
Predictive performance of mechanistic and predictive biomarkers HSF1, MARK3, and RBCK1. Receiver operating characteristic (ROC) curves from univariate logistic regression models built using normalized expression data to distinguish between *in silico* good and poor responders. Cross-validated area under the curve (AUC) values are reported for each biomarker.

To assess their ability to distinguish between good and poor *in silico* responders to regorafenib, we constructed univariate logistic regression models using normalized expression data. HSF1 and MARK3 showed limited predictive performance, with cross-validated AUC values below 0.7. In contrast, RBCK1 achieved a cross-validated AUC of 0.7, indicating moderate predictive value (see Figure 6).

## 4 Discussion

This study presents an *in silico* clinical trial framework designed to simulate first-line regorafenib treatment in elderly patients with mCRC, a population often underrepresented in clinical research despite their high disease burden. By integrating transcriptomic data into a systems biology-based modeling approach (TPMS), we developed 399 digital patient models that enabled the prediction of drug-target based response and the identification of mechanistic and predictive biomarkers.

A key strength of this work is the use of large, publicly available transcriptomic datasets combined with cross-platform normalization techniques to assemble a representative discovery cohort of untreated elderly mCRC patients. This cohort provided the foundation for building individualized protein expression profiles—referred to as IDEs (individual differential expressions)—that capture the unique molecular features of each patient relative to healthy controls. Notably, these IDEs reflected known population-level cancer signatures, underscoring their biological relevance.

We employed the TPMS systems biology modeling approach (Jorba et al., 2020; Segu-Verges et al., 2021) to simulate the effect of the inhibition of regorafenib targets in a mCRC knowledge set. This simulation yielded a distribution of treatment responses, quantified as TSignal values. Based on this distribution, we defined the top and bottom 10% of models as good and poor responders, respectively. By comparing the MoAs between these two groups, we identified 213 mechanistic biomarkers—proteins whose activation profiles distinguished good from poor responders.

Enrichment analysis of these biomarkers revealed biologically plausible pathways. Proteins more active in poor responders were enriched in pathways previously associated with resistance to treatment, including PI3K-Akt (Yang et al., 2023), NOD-like receptor (Canning et al., 2015; Shen et al., 2025) and Toll-like receptor signaling (Sasaki et al., 2020; Papadakos et al., 2023). These pathways are known to mediate survival and immune evasion mechanisms that can diminish therapeutic efficacy. Conversely, good responders showed enrichment in DNA repair pathways—though their role in modulating treatment response remains context-dependent (Kiwerska and Szyfter, 2019).

To identify predictive biomarkers, we compared the IDEs of good and poor responders and found 173 proteins with significant differential expression patterns. Importantly, many predictive candidates had a close relationship (one link connection) with the mechanistic set, with four proteins—HSF1, MARK3, LHCGR, and RBCK1—shared across both. Biomarkers with both mechanistic and predictive relevance are particularly valuable, as they are more likely to reflect true drug-response biology rather than statistical artifacts (Robinson et al., 2013).

Further analyses focused on these four candidate biomarkers. In the independent validation cohort, the entire modeling procedure was replicated, and HSF1, MARK3, and RBCK1 consistently retained their mechanistic activity profiles, reinforcing their biological relevance. Moreover, all three proteins were linked to previously reported regorafenib-associated predictive miRNAs. Notably, even under more stringent criteria for defining miRNA–target relationships, the association with RBCK1 remained robust. While these miRNA–target links provide additional support for the involvement of these proteins in modulating treatment response, we acknowledge that such associations remain putative in the absence of functional validation. Nonetheless, the consistency of these associations across datasets supports their value as hypothesis-generating leads for future mechanistic studies. Moreover, RBCK1 was the only candidate to demonstrate moderate predictive power (cross validated AUC = 0.7) in univariate logistic regression models using transcriptomic data.

RBCK1 encodes a ubiquitin ligase involved in transcriptional regulation and immune signaling and is known to modulate NF-κB pathways, which are often implicated in tumor progression and drug resistance (see below). In our study, RBCK1 was significantly overexpressed in mCRC samples compared to healthy controls, and its elevated expression was strongly associated with poor response to regorafenib. Importantly, *in silico* simulations indicated that regorafenib does not modulate RBCK1 activity, suggesting that its persistent activation may contribute to resistance mechanisms. One possible explanation is that RBCK1-driven signaling bypasses regorafenib’s primary inhibitory targets, sustaining pro-survival pathways that diminish drug efficacy. Prior studies have implicated RBCK1 in chemotherapy resistance in colorectal (Liu et al., 2019), liver (Chen et al., 2022) and ovarian (Wang et al., 2022) cancers. A recent study also demonstrated experimentally that RBCK1 can significantly inhibit the apoptosis and promote invasion in hepatocellular carcinoma, supporting its role in aggressive tumor behavior (Yu et al., 2024). A conceptual parallel exists where RBCK1 promotes resistance to the tyrosine kinase inhibitor sunitinib in clear cell renal cell carcinoma (Wang et al., 2023), further validating its potential as a treatment-refractory biomarker. While these findings are consistent with known resistance mechanisms, further experimental validation is needed to clarify RBCK1’s downstream effectors and its direct role in limiting regorafenib efficacy.

In conclusion, our study identifies RBCK1 as a promising biomarker of poor response to regorafenib in elderly mCRC patients. Its consistent activity across modeling, expression, and validation analyses underscores its potential as both a prognostic and mechanistic marker of resistance. Although the predictive performance based on transcriptomic data is modest (AUC ∼0.7), which limits immediate clinical applicability, the reproducibility of our mechanistic findings—supported by prior evidence in other cancer types—suggests that RBCK1 may serve as a valuable component of a broader predictive framework. To enhance clinical translatability, future efforts should explore more practical detection methods (e.g., immunohistochemistry or ELISA-based assays) and assess the utility of integrating this biomarker into multivariable models that combine biomarker levels with relevant clinical parameters.

Integrating such computational frameworks into early phases of clinical development could accelerate biomarker discovery and optimize treatment strategies for difficult-to-study patient populations.

## Data Availability

The results of the digital models of the 399 mCRC patients modelling the disease state and regorafenib treatment have been uploaded to Zenodo (10.5281/zenodo.15654233).
