# Supplementary material for: Digital patient modeling identifies predictive biomarkers of regorafenib response in elderly metastatic colorectal cancer
Source: Front Syst Biol. 2025 Sep 15;5:1648559. doi: 10.3389/fsysb.2025.1648559 (PMC12477164; doi:10.3389/fsysb.2025.1648559)
Supplement: Supplementary file 1 [file Presentation1.pdf]

Supplementary Figures

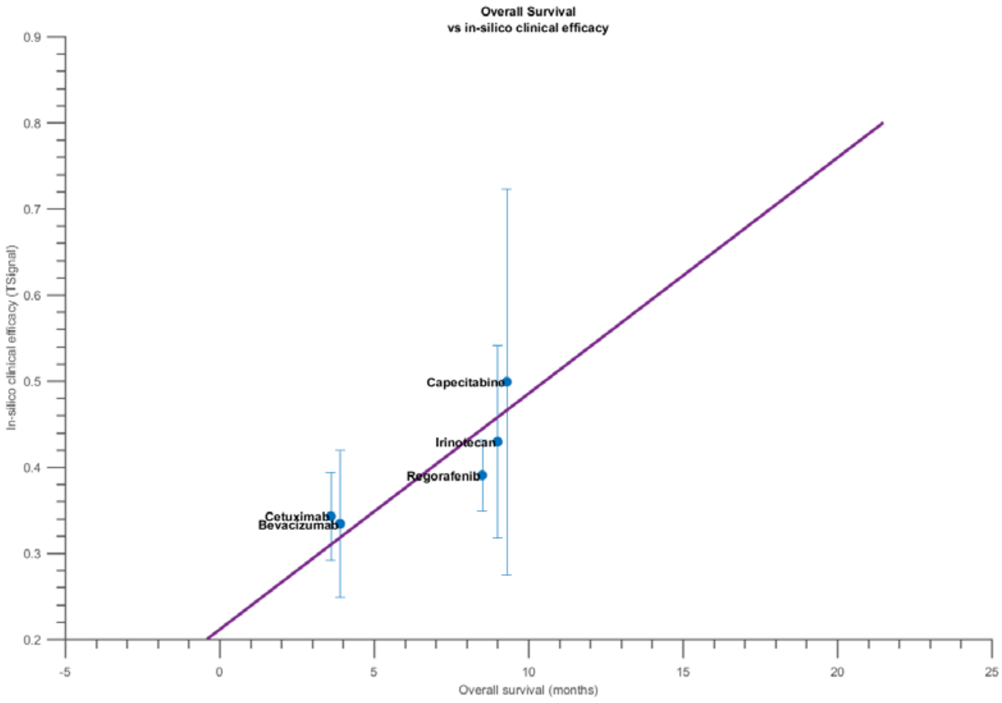

Supplementary Figure 1. Calibration of *in silico* signal (TSignal) with overall Survival (OS). In-silico efficacy measured for 5 drugs using the TPMS modeling approach against mCRC protein knowledge set. Depicted are the final values over the mCRC protein knowledge set that has been optimized to get the best adjustment possible with OS. Pearson correlation coefficient of 0.87.

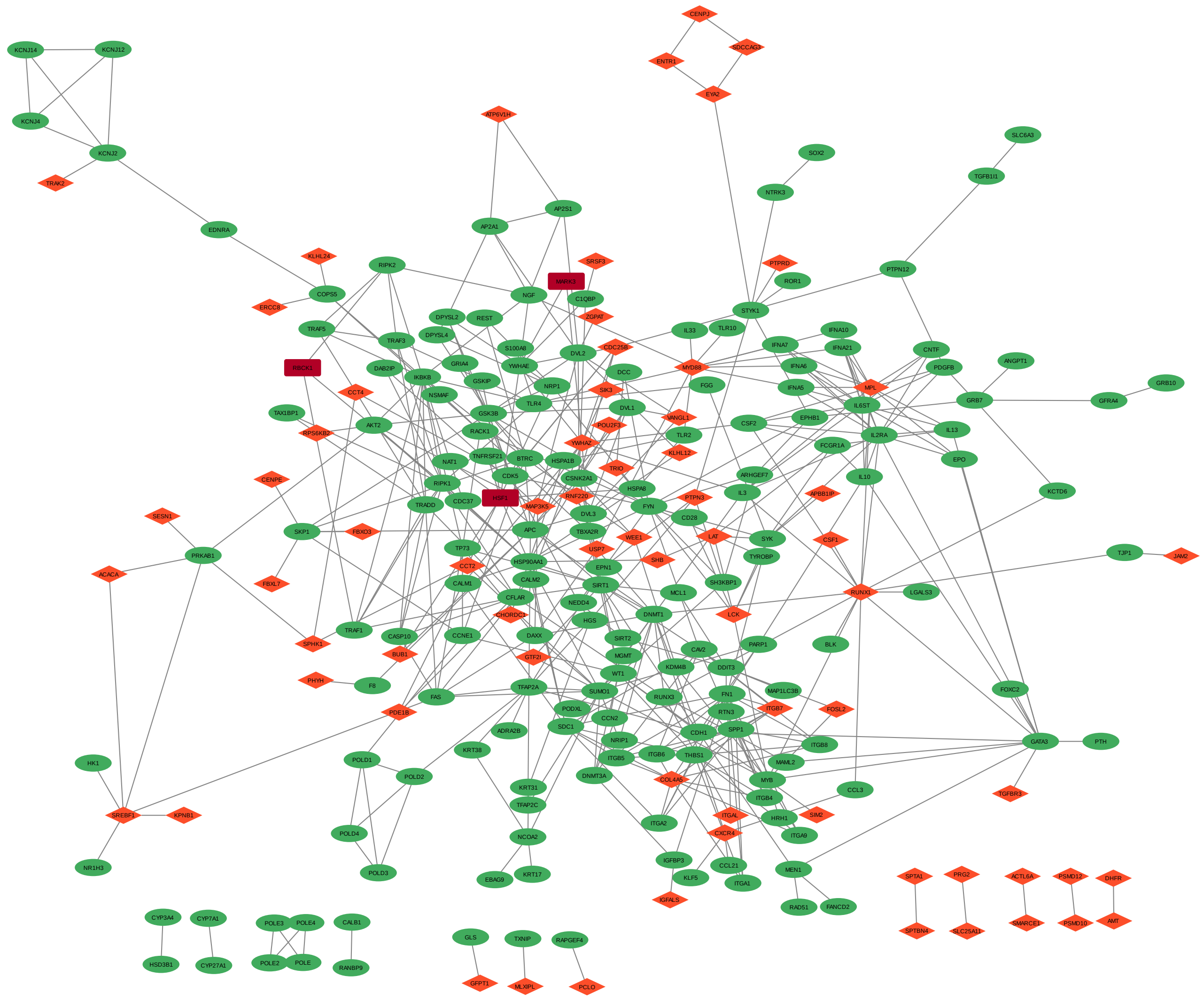

Supplementary Figure 2: Association between mechanistic and predictive biomarkers identified in the discovery mCRC population. Mechanistic biomarkers (213) are depicted as green oval nodes. Predictive biomarkers (173) are depicted as orange diamond-shaped nodes. Proteins both mechanistic and predictive biomarkers – HSF1, MARK3 and RBCK1 – are shown as red rectangular nodes (LHCGR is not shown as it did not have any interaction with the 173 predictive biomarkers).

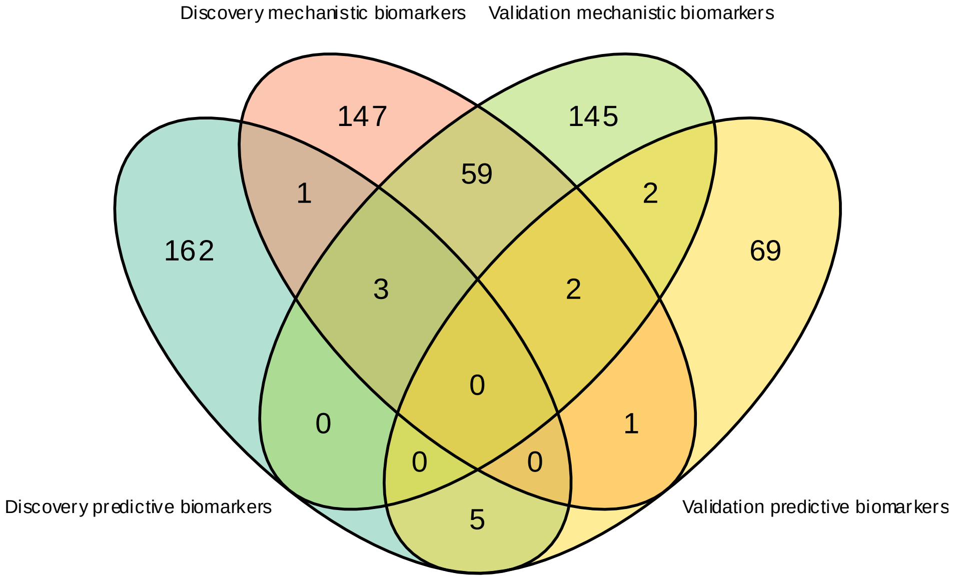

Supplementary Figure 3: Venn diagram between the four biomarker lists identified in the discovery and validation mCRC populations.
